# Cultural Adaptation of Together+, a Status-Neutral mHealth Intervention to Improve HIV Prevention and Care for Adolescent and Young Men Who Have Sex With Men in Vietnam: Protocol for a Co-Design Study

**DOI:** 10.2196/73895

**Published:** 2025-09-23

**Authors:** Minh X Nguyen, William C Miller, Le Minh Giang, Patrick S Sullivan

**Affiliations:** 1 Department of Epidemiology Institute of Preventive Medicine and Public Health Hanoi Medical University Hanoi Vietnam; 2 Center for Training and Research on Substance Abuse - HIV Hanoi Medical University Hanoi Vietnam; 3 Department of Epidemiology Gillings School of Global Public Health University of North Carolina at Chapel Hill Chapel Hill, NC United States; 4 Department of Epidemiology Rollins School of Public Health Emory University Atlanta, GA United States

**Keywords:** mobile health, mHealth, adolescents and young men who have sex with men, Vietnam, HIV, status neutral

## Abstract

**Background:**

Adolescent and young men who have sex with men (AYMSM) are experiencing an ongoing epidemic in Vietnam. HIV testing and preexposure prophylaxis uptake among AYMSM remain low in Vietnam, especially compared to older men. AYMSM living with HIV are also less likely to initiate HIV care. M-Cubed is a status-neutral mobile intervention developed in the United States focusing on HIV prevention and care among men who have sex with men. The app has the potential to significantly contribute to improving the HIV prevention and care continuum for AYMSM.

**Objective:**

We propose to adapt the M-Cubed app for AYMSM in Vietnam to create Together+, a status-neutral app that promotes HIV testing, preexposure prophylaxis use, and HIV care.

**Methods:**

Adaptation will focus on ensuring that the content, features, and design of the app are culturally relevant to AYMSM in Vietnam. The adaptation process will comprise five phases: (1) adaptation and creation of videos and messages in Vietnamese, (2) in-depth interviews to further inform app adaptation, (3) app prototype development, (4) app theater testing, and (5) beta testing of the adapted app. AYMSM aged between 15 and 19 years and health care staff in Hanoi, Vietnam, will be recruited for in-depth interviews in phase 1 and focus group discussions in phase 4. Qualitative data will be analyzed thematically, and results will be generated after reviewing memos, code reports, and the matrix. To evaluate the feasibility and usability of the Together+ app, we will provide access to 30 AYMSM and encourage them to use the app for 30 days. We will assess observed use and collect quantitative and qualitative data from test users. After 30 days, we will evaluate the usability and feasibility of the Together+ app through in-app analytics as well as online quantitative surveys and individual exit interviews with participants.

**Results:**

As of September 2025, we are in the process of adapting the set of 15 videos for Vietnamese AYMSM (phase 1) and analyzing qualitative data from in-depth interviews (phase 2). Data collection for the pilot phase will be completed by August 2026.

**Conclusions:**

Adaptations of proven effective interventions are a promising and efficient way to develop interventions for new service populations but require formal adaptation and evaluation in the new service population. Once culturally adapted for AYMSM in Vietnam, the Together+ app has the potential to significantly contribute to improving the HIV prevention and care continuum for this population. The findings of the adaptation process will document the level of usability and feasibility of the Together+ app and shed light on the perceptions of AYMSM and other stakeholders in Vietnam regarding status-neutral mobile health interventions.

**International Registered Report Identifier (IRRID):**

PRR1-10.2196/73895

## Introduction

### Background

The Asia-Pacific region ranks second highest among all regions in new diagnoses of HIV infection. The region accounts for almost a quarter of all new cases globally [1]. More than 90% of the people with HIV live in 6 countries in this region, including Vietnam [2]. In Vietnam, new HIV cases are primarily among men who have sex with men (MSM), among whom the HIV prevalence has more than doubled in the last 5 years [3]. Many of the newly identified cases in Vietnam are among adolescent and young MSM (AYMSM), and the prevalence of HIV among them is 12% [4]. In response to this epidemic among AYMSM, the Vietnamese government has recently allowed adolescents aged between 15 and 17 years to get tested for HIV without parental consent [5]. However, HIV testing and preexposure prophylaxis (PrEP) uptake among AYMSM in Vietnam have remained low; 58% of the AYMSM had never been tested for HIV, and 63% had never used PrEP before [6]. When compared with older MSM, AYMSM were also less likely to initiate HIV care promptly after HIV diagnosis [7]. Therefore, specific interventions are needed to reach the unique subgroup of AYMSM with HIV prevention and care services.

**Figure 1:**
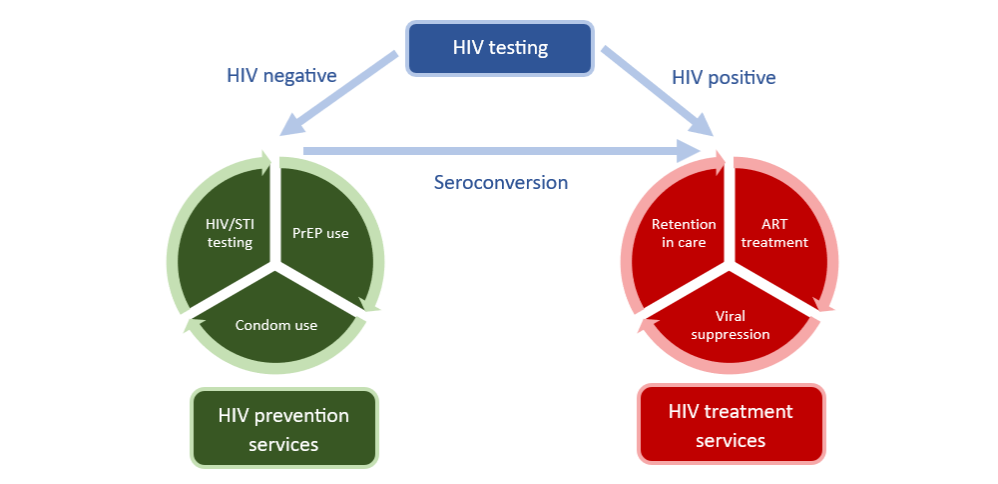
HIV status-neutral approach (adapted from US Centers for Disease Control and Prevention Issue Brief [8]). This figure illustrates a status-neutral HIV care framework. Individuals who test negative for HIV are linked to prevention strategies such as preexposure prophylaxis (PrEP), condom use, and sexually transmitted infection (STI) testing. Those who test positive for HIV are connected to antiretroviral (ART) treatment, viral suppression, and retention in care.

To end the HIV epidemic, people with and without HIV must be aware of and have access to treatment and prevention options (Figure 1 [8]). The status-neutral approach bridges the divide between HIV treatment and prevention [8]. This approach is advantageous for several reasons [8,9]: (1) HIV status-neutral interventions encompass testing, prevention, and treatment; therefore, common barriers to HIV testing; PrEP use; and HIV care, including difficulties navigating the health system, structural stigma, and health inequity, can be overcome [8,9]; (2) combining HIV prevention and treatment tools can create a unified setting that makes it more convenient for a person to access a comprehensive range of essential services for HIV testing, prevention, and treatment [8]; and (3) health equity may be improved by normalizing and destigmatizing HIV testing, PrEP use, and treatment [9].

The use of mobile health (mHealth) is a promising approach for youth in low- and middle-income countries (LMIC), who are usually more technology savvy than older generations [10]. Many AYMSM are avid users of smartphones and might prefer receiving HIV-related information in a familiar and discreet environment [11,12]. The advantages of mHealth interventions include easy access, privacy, low cost, and the ability to reach hidden populations [10]. In Vietnam, 89% of youth considered themselves as having intermediate or advanced levels of mobile phone proficiency, and 81% had used a health care app before [13]. In a survey of MSM in Vietnam, 92% reported owning a smartphone, and 75% reported using their smartphones to find information related to HIV or sexually transmitted infections (STIs) online [14]. Nevertheless, mHealth interventions have rarely been developed specifically for AYMSM in LMIC [15,16]. For example, in a systematic review, we found that fewer than 20% of technology-focused intervention studies for MSM were conducted in LMIC, and fewer than 10% of the studies focused on young MSM (all of which were conducted in the United States) [16].

To end the HIV epidemic, people with and without HIV must be aware of and have access to treatment and prevention options. People of either serostatus are not distinct populations but rather 1 group with similar medical and social service needs [8]. The status-neutral approach in HIV care is a public health strategy that provides a unified pathway to care regardless of whether a person is living with HIV or at risk of acquiring HIV. This approach is designed to bridge the divide between HIV treatment and prevention [8]. Status-neutral mHealth interventions might offer unique opportunities to reach AYMSM in these settings. To the best of our knowledge, no apps that support both HIV prevention and care for AYMSM have been specifically designed and tested in Vietnam. In this study, we will adapt M-Cubed, an evidence-based intervention (EBI) developed in the United States, to create the Together+ app. We selected M-Cubed to adapt for AYMSM in Vietnam because the platform app on which M-Cubed was developed has high acceptability and feasibility [17]. In a randomized trial conducted by Sullivan et al [18], it was concluded that MSM who used the M-Cubed app were approximately twice as likely to use PrEP and undergo HIV testing compared to those who did not. The M-Cubed app is listed in the Compendium of EBIs and Best Practices for HIV prevention by the US Centers for Disease Control and Prevention (CDC) [19].

### Aims

We conduct this study with the following aims: (1) to understand barriers to and facilitators of HIV testing, PrEP use, and HIV care among AYMSM in Vietnam; (2) to explore perspectives and preferences of AYMSM and other key stakeholders for status-neutral mHealth interventions to promote HIV testing, PrEP use, and HIV care among AYMSM in Vietnam; and (3) to adapt the M-Cubed app to develop a status-neutral mHealth intervention to promote HIV testing, PrEP use, and HIV care among AYMSM in Vietnam. We hypothesize that AYMSM and key stakeholders will express interest in a status-neutral mHealth intervention. We also hypothesize that the M-Cubed app can be successfully adapted to the Vietnamese context and will be perceived by AYMSM as acceptable, culturally appropriate, and feasible to use for promoting HIV testing, PrEP uptake, and linkage to HIV care, regardless of HIV status.

## Methods

### Study Design and Setting

**Figure 2:**
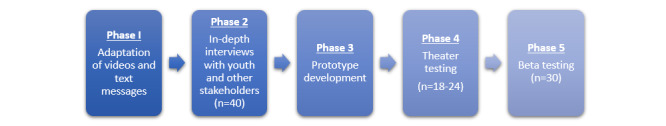
Study phases. This figure outlines the phased process of adapting and testing the Together+ app.

We will conduct this study in Hanoi, the capital of Vietnam. Hanoi has the largest estimated MSM population in the country, with more than 30,000 MSM [20]. We will collaborate with a local app developer to tailor the M-Cubed app to address the specific needs of AYMSM related to HIV testing, PrEP use, and HIV care. Adaptation will focus on ensuring that the concepts, language, and context of the app are culturally relevant for AYMSM in Vietnam. The adaptation process will comprise five main phases: (1) adaptation and creation of videos and messages in Vietnamese, (2) in-depth interviews (IDIs), (3) prototype development, (4) theater testing, and (5) beta testing (Figure 2).

This study uses a co-design approach by actively involving AYMSM throughout the adaptation process. To understand the preferences and needs of AYMSM for status-neutral mHealth interventions and enhance the cultural relevance of the app, AYMSM and health care workers in Vietnam will be engaged throughout the adaptation process using IDIs, focus group discussions (FGDs), and community advisory board (CAB) meetings.

### The Original App

The M-Cubed app is a status-neutral mobile intervention for HIV prevention and care needs of MSM [18]. It was developed and tested in the United States and is based on the social cognitive theory [21]. The intervention offers a range of HIV testing, prevention, and care services. Key features of the app include self-screening for HIV and STI risk, a scheduling and reminder system for HIV and STI testing, a PrEP and postexposure prophylaxis eligibility screener, an ordering platform for delivery of at-home test kits and condoms, and locators for HIV services [18] (Table 1). The M-Cubed app also provides written and video messages customized to the participant’s risk, HIV status, and corresponding needs [18].

**Table 1:** Key features of the M-Cubed app.

	Key features
Initial and monthly risk assessments	Tailored, HIV-related prevention suggestions based on quiz responses
PrEP^a^ screener	Assess PrEP eligibility using a questionnaire
nPEP^b^ screener	Assess nPEP eligibility using a questionnaire
Find my frequency	Suggest HIV testing frequency based on risks
Compare HIV tests	Comparison and recommendation on HIV tests
My test plan	Users can plan an HIV test by date, time, and location
Reminders	Reminders to take PrEP or get tested for HIV
Ordering	Free at-home HIV test kits, condoms, and lubricants
Location details and map	Provide a map and details about testing locations
Written and video messages	Focus on condom use, HIV and STI^c^ testing, PrEP use, condom use, ART^d^ use, and engagement in care
FAQs^e^	Frequently asked questions related to HIV

^a^PrEP: preexposure prophylaxis.

^b^nPEP: nonoccupational postexposure prophylaxis.

^c^STI: sexually transmitted infection.

^d^ART: antiretroviral therapy.

^e^FAQ: frequently asked question.

### Community Advisory Board

The study team has established a CAB consisting of 8 members (n=4, 50% AYMSM with both HIV statuses; n=4, 50% HIV health care workers, ie, physicians, nurses, and counselors). The app’s name (Together+) was suggested by the CAB to reflect the app’s inclusive approach to supporting all AYMSM regardless of their HIV status. Both the word “Together” and the “+” sign emphasize unity, community, and shared experiences of users of both HIV statuses. We will meet with CAB members regularly to ensure that the contents of Together+ follow current Vietnamese guidelines for HIV testing, PrEP initiation, and HIV care and include information about HIV and PrEP clinics in Hanoi. We will also consult with them about the social, cultural, and legal contexts of HIV service delivery in Vietnam.

### Study Phases

#### Phase 1: Adaptation and Creation of Videos and Messages in Vietnamese

The original M-Cubed app has 15 videos with a total duration of 23 minutes. These are CDC-made, public service announcements developed in the United States. The videos have 5 domains: condoms, PrEP, HIV testing, STI testing, and antiretroviral therapy use or engagement in care. The study team will collaborate with the CAB to review the content of these videos and make necessary changes to ensure that they are culturally relevant to the current HIV landscape in Vietnam. The CAB will provide feedback on the appropriate video formats for each type of content, including live-action videos and animated and motion-graphic videos, and content not included in the original set of videos, such as event-driven PrEP, long-acting injectable PrEP, and Undetectable=Untransmittable (U=U) message, will be added. A new set of 15 videos in Vietnamese, featuring Vietnamese actors, will be created. Text messages will be translated into Vietnamese and refined by the study team and the CAB to fit the local context.

#### Phase 2: IDIs With AYMSM and Other Stakeholders

We will recruit up to 40 participants from 3 groups for IDIs: AYMSM with HIV (n≈15); AYMSM with unknown HIV status or without HIV (n≈15); and key stakeholders (n≈10), including health officials and staff from HIV and PrEP clinics. A trained Vietnamese interviewer will ask participants about their perspectives on the acceptability, feasibility, and potential impacts of the Together+ app to improve HIV testing, PrEP use, and HIV care for AYMSM in Vietnam. The IDI guide is included in Multimedia Appendix 1. Key features of the original app will be summarized, and participants will share their opinions on desired contents, features, and design of the Together+ app. Feedback and suggestions for the set of videos and messages will also be collected during the IDIs.

#### Phase 3: Prototype Development

On the basis of input from the CAB members and the results of the IDIs, we will summarize data on the preferred content, features, and design of the app and use those summaries for discussion with a local app developer to create the prototype of the app. Prototype development is an iterative procedure where the study team will oversee the process and conduct weekly meetings with the app developer. During these meetings, we will provide an update on the progress, share materials, discuss any issues, and refine the prototype before using it for theater testing.

#### Phase 4: Theater Testing

When the prototype is available, theater testing will be conducted through 3 FGDs (6-8 participants each), 2 for AYMSM and 1 for health care workers who care for AYMSM. During theater testing, participants will interact with the Together+ prototype and provide feedback on the functionality, appearance, and usability of the platform. During the FGD with AYMSM, the interviewer will ask participants to identify the components of the app that they like or dislike and features that could facilitate app engagement among youth. The FGD with other stakeholders will aim to understand their perspectives on the HIV and PrEP contents to be included and aspects of the app that could be improved to encourage HIV testing and PrEP uptake among AYMSM. The FGD guide is included in Multimedia Appendix 2. Data will be used to refine the app and create the beta version.

#### Phase 5: Beta Testing

Even though the platform on which the M-Cubed app was developed has been proven to be highly acceptable and feasible [17], beta testing will be conducted to ensure that the Together+ app is usable and its features are used as intended by the target population. A total of 15 AYMSM living with HIV and 15 not living with HIV or having unknown HIV status will be invited to participate in beta testing. Study staff members will be trained on the contents and features of the app beforehand. During the study visit, staff members will help participants download the app and provide instructions on how to use it. Participants who provide consent to download the app on their own will be encouraged to use the app for 30 days and take advantage of all features that are of interest to them. After 30 days, we will evaluate the usability and feasibility of the Together+ app through an online quantitative survey and individual exit interviews with each participant.

### Participants and Recruitment Strategy

We will recruit AYMSM and other stakeholders into the study. Recruitment methods for AYMSM will include posting advertisements on popular social networking websites (eg, Facebook and Instagram), distributing flyers about the study, and direct outreach at community-based organizations and clinics serving MSM and people with HIV in Hanoi. Other stakeholders are health care workers who will be recruited through referral and direct outreach to Hanoi CDC and HIV and PrEP clinics in Hanoi. Interested participants will be referred to the study staff for eligibility screening over the phone. During phase 2 (IDIs) and phase 4 (theater testing), eligibility criteria for AYMSM include the following: (1) aged between 15 and 19 years, (2) biologically male at birth, (3) identifying as male, and (4) having previously engaged in anal sex with another man. We will purposively sample an equal number of AYMSM from 2 groups: AYMSM with HIV-negative or unknown status and AYMSM with HIV-positive status. In addition, AYMSM participating in theater and beta testing are required to own a smartphone. For beta testing, AYMSM will be ineligible if they are unwilling to provide contact information or plan to leave Hanoi in the next 3 months.

Eligibility criteria for other stakeholders include the following: (1) health officials working in HIV prevention or HIV care at Hanoi CDC for at least 6 months or (2) counselors or health care providers working in HIV and PrEP clinics in Hanoi for at least 6 months.

### Data Collection

Before IDIs and FGDs, basic demographic characteristics will be collected from each consenting respondent. IDIs and FGDs will be conducted in a private room by trained interviewers and will be audio recorded. During beta testing, we will assess the usability of the adapted app using the System Usability Scale (SUS) through an online survey sent to participants. The SUS is a validated 10-item measure that has been extensively used in mHealth research to measure the acceptability of mobile apps [22]. Feasibility will be evaluated based on data on app engagement collected through app analytics, such as the number of times participants accessed the app over 30 days, features used, and duration of use. Beta testers will be interviewed online at the end of this phase to provide feedback on app functionality, technical performance, errors, and overall experiences using the app. This in-depth exit interview will be completed on Zoom (Zoom Communications, Inc) by a trained interviewer. All interviews will be audio recorded and transcribed.

### Data Analysis

For qualitative data in phases 2 and 4, memos of emergent themes and patterns will be written for each interview. A codebook will be developed based on the main topics, and the coding process will include 2 investigators to ensure intercoder reliability. A qualitative matrix will be created to document similarities and differences in the perspectives of participants. Study results will be generated after reviewing memos, the codebook, and the matrix.

After beta testing, we will summarize the mean SUS score as well as app use data to evaluate usability and feasibility, respectively. Data from the SUS will be scored from 0 to 100, and a score of 50 or greater indicates that the app is acceptable [23]. We will apply a rapid qualitative approach to summarize and analyze data from exit interviews at the end of the beta testing phase. For each question in the interview guide, the notetaker will take real-time summary descriptions of participant responses in Vietnamese, using a template of the interview, consistent with previously described approaches for generating interview summaries in real time [24,25]. Two investigators will use matrix or table-based analysis methods to summarize major discussion points across the templated summaries [24,25]. Rigor and validity will be established by independently coding and assigning these data in the matrix, along with discussions with the larger team. These data will be used to refine the beta testing for further and larger-scale evaluation of the adapted app.

### Sample Size

Following recommendations for qualitative studies, a sample size of 30 to 40 participants for the qualitative study would likely be sufficient for saturation [26]. In addition, our sample size for the adaptation phases is similar to other pilot studies involving the development of mHealth interventions for young MSM [27,28]. The inclusion of different groups of participants in our study allows for the exploration of various perspectives from different types of stakeholders.

### Ethical Considerations

This study has been reviewed and approved by the institutional review board at Hanoi Medical University (1555/GCN-HMUIRB). All research activities involving human participants complied with institutional and national ethical guidelines.

Participants will provide written informed consent before participation. For participants aged between 15 and 17 years, assent will be obtained, and parental consent will be waived. All participants will be informed of their right to withdraw from the study at any time without any consequences.

Data collected during the study were deidentified at the point of entry and stored securely on password-protected, encrypted servers accessible only to authorized research personnel. No identifiable images or information are presented in this paper or the multimedia appendices.

Participants will receive compensation of VND 300,000 (US $11.4) to VND 500,000 (US $19) to cover transportation and time spent during study participation. This amount was determined to be aligned with local standards for ethical participant compensation.

## Results

As of September 2025, we are implementing phase 1 and 2of the study. The study team has conducted 3 meetings with the CAB, and we plan to conduct meetings with them every 3 months. We are in the process of adapting the set of 15 videos for Vietnamese AYMSM (phase 1). We have finished recruiting AYMSM and other stakeholders for IDIs and we are transcribing and analyzing these data (phase 2). Data collection for the last phase will be completed by August 2026, and study results are expected to be available by the end of 2026.

## Discussion

### Anticipated Findings

AYMSM in Vietnam and similar low-resource countries bear disproportionate burdens of HIV. This population urgently requires attention and intervention. AYMSM have unique needs for HIV prevention and care due to the major physical and emotional changes that they undergo during adolescence [29]. We hypothesize that AYMSM in Vietnam will identify both structural (eg, stigma and limited access) and individual-level (eg, low-risk perception and mental health challenges) barriers to HIV testing, PrEP use, and HIV care while also recognizing facilitators of HIV care, such as peer support, digital health tools, and youth-friendly services. We also hypothesize that AYMSM will prefer features that offer privacy, tailored health information, motivational messaging, and easy linkage to youth-friendly HIV services in the status-neutral app.

This study addresses the urgent need for culturally tailored, accessible, and effective HIV prevention and treatment solutions for AYMSM in Vietnam—a population with high HIV prevalence that is underserved in traditional health care settings. To improve the HIV prevention and care continuum for AYMSM, we are creating the culturally adapted Together+ app. The findings of this study will assess the usability and feasibility of the Together+ app and shed light on perceptions of AYMSM and other stakeholders in Vietnam on status-neutral mHealth interventions.

Our approach builds on evidence from the global literature that mHealth interventions can significantly enhance HIV prevention behaviors among key populations [10]. Previous work with MSM in high-income settings has demonstrated that tailored, mobile-based interventions improve engagement with HIV testing and PrEP uptake [18,30]. In addition, our use of a status-neutral framework, which emphasizes services for individuals regardless of HIV status, aligns with emerging global public health strategies that aim to reduce stigma and fragmentation in care delivery [31,32]. The status-neutral approach facilitates the simultaneous management of health care and social service needs of all people affected by HIV. This approach is relatively novel in Vietnam and contributes to a growing body of work promoting more inclusive, person-centered care models.

Given the ubiquity of smartphones among AYMSM and the effectiveness of mHealth interventions on HIV-related outcomes, our status-neutral app will have the potential to reach and benefit this vulnerable population. We opted to adapt an existing effective app rather than design a new app from scratch because adapting EBIs for low-resource settings is more efficient than de novo development. Lessons learned and other data from the adaptation process can be disseminated and applied to the adaptation of other technology-based interventions in LMIC.

### Strengths and Limitations

This study exemplifies a co-design approach by actively engaging AYMSM and health care workers in shaping the creation of the Together+ app. Rather than making top-down modifications based solely on theory and expert opinion, the process integrates user insights at multiple stages, from content creation to theater and beta testing, ensuring that the app is culturally relevant and meets the specific needs of AYMSM in Vietnam. Collaboration with a local app developer further supports this participatory approach, allowing for iterative refinements based on user feedback. By incorporating diverse perspectives through IDIs, FGDs, and CAB meetings, we will foster a sense of ownership among stakeholders, ultimately enhancing the app’s usability and effectiveness.

Due to the limited timeline and funding received by this research, we could only conduct field testing of the Together+ app in a sample of AYMSM in 1 city in Vietnam. We may experience challenges recruiting AYMSM with HIV, but we expect to be able to enroll our small sample size, especially given our experience of recruiting AYMSM. If enrollment challenges arise, we will partner with community-based organizations, HIV clinics, and peer networks while leveraging social media outreach to improve recruitment rates among AYMSM with HIV. In addition, we will ensure confidentiality of participants and consult with the CAB to build trust with and encourage participation from AYMSM.

### Future Directions and Dissemination Plans

Building on the results of this formative study, we plan to conduct a larger, multisite randomized controlled trial to assess the effectiveness of the Together+ app in improving HIV prevention and care outcomes among AYMSM. Future studies should also investigate the app’s impact on mental health, sexual well-being, and social connectedness, which are often interconnected with HIV-related behaviors in this population.

In parallel, we will disseminate lessons learned from the adaptation process to support other researchers and practitioners aiming to adapt technology-based HIV interventions in LMIC contexts. By advancing the science of culturally informed, status-neutral, and youth-centered mHealth design, this study contributes to global efforts to reduce HIV incidence and improve care engagement among vulnerable populations.

### Conclusions

This protocol details the adaptation process of the M-Cubed app to create the Together+ app, a version for use among AYMSM in Vietnam. By adapting an evidence-based status-neutral mHealth intervention from the United States, this research ensures that the contents, features, and design of the app align with the unique social, cultural, and structural factors influencing HIV risk and care in Vietnam. Findings will inform the planning for a future hybrid effectiveness-implementation randomized controlled trial to rigorously test the effectiveness of the Together+ app and explore factors affecting its scaling up in Vietnam.

## Appendix

Multimedia Appendix 1 In-depth interview guide.

Multimedia Appendix 2 Focus group discussion guide.

Multimedia Appendix 3 Peer review report by Collaborative Initiative for Paediatric HIV Education and Research (CIPHER) Grant Review Committee, International AIDS Society (IAS).

## Abbreviations

AYMSM: adolescent and young men who have sex with men
CAB: community advisory board
CDC: Centers for Disease Control and Prevention
EBI: evidence-based intervention
FGD: focus group discussion
IDI: in-depth interview
LMIC: low- and middle-income country
mHealth: mobile health
MSM: men who have sex with men
PrEP: preexposure prophylaxis
STI: sexually transmitted infection
SUS: System Usability Scale

## Data Availability

Data sharing is not applicable to this paper as no datasets were generated or analyzed during this phase of the study.
