# Supplementary material for: Cultural Adaptation of Together+, a Status-Neutral mHealth Intervention to Improve HIV Prevention and Care for Adolescent and Young Men Who Have Sex With Men in Vietnam: Protocol for a Co-Design Study
Source: JMIR Res Protoc. 2025 Sep 23;14:e73895. doi: 10.2196/73895 (PMC12504892; doi:10.2196/73895)
Supplement: Multimedia Appendix 1 [file resprot_v14i1e73895_app1.docx]

**In-Depth Interview Guide**

**Moving towards a status-neutral approach to improve HIV testing, prevention and care for adolescent and young men who have sex with men in Vietnam: adaptation of an evidence-based mobile health intervention**

**INTRODUCTION**

**In-Depth Interview**

Thank you for agreeing to meet with me today. My name is [introduce yourself and your role]. I’m working with a team of researchers from Hanoi Medical University.

We aim to better understand the barriers to HIV testing, PrEP use, and HIV treatment, as well as your perspective on a status-neutral HIV intervention designed to support HIV testing, treatment, and PrEP uptake among adolescent and young MSM.

Your feedback will help us design an ideal intervention for AYMSM in Vietnam, with the goal of preventing HIV transmission among HIV-negative individuals and increasing HIV treatment uptake among those living with HIV.

Everything you share with us will be kept confidential by the research team. Our conversation will be audio-recorded and transcribed, but your name or any identifying information will not be linked to anything you say. The recordings will be destroyed after analysis is complete.

There are no right or wrong answers. I may ask you to elaborate on something you've said, but feel free to skip any questions you're uncomfortable with. Occasionally, I might move on to another question to ensure we cover all topics during our interview today.

Do you have any questions for me before we begin?

**Interview Objectives**

- Understand the barriers and facilitators to HIV testing and PrEP use among AYMSM
- Explore participants’ perspectives on AYMSM’s preferences for a status-neutral HIV mHealth app
- Gather participants’ feedback on messages and videos for the app

**Opening Question**

**Can you tell me a little bit about yourself?**
*Prompts: How old are you? Please tell me about your typical day.*

**Part 1: Barriers and Facilitators to HIV Testing, PrEP Use, and HIV Treatment**

*(Suggested time: 35–40 minutes)*

1. **What makes HIV testing easier for adolescent and young MSM? Please explain.**
   Prompts:
   - Personal factors: risk perception, knowledge of HIV
   - Interpersonal factors: influence from friends, partners, family
   - Social factors: school education, public attitudes
   - Organizational factors: access to health services, cost
2. **What makes HIV testing more difficult or discouraging for AYMSM? Please explain.**
   Prompts:
   - Under 18, low perceived risk, limited HIV knowledge, mental health
   - Peer/family influence, social stigma, lack of information
   - Difficulty accessing services, high costs
3. **What makes PrEP use easier for AYMSM? Please explain.**
   Prompts:
   - PrEP-related: ease of use, adherence
   - Personal: risk awareness, PrEP knowledge
   - Peer/family influence, school messaging, access and affordability
4. **What makes PrEP use more difficult for AYMSM? Please explain.**
   Prompts:
   - PrEP-related: side effects, adherence
   - Under 18, low risk perception, limited knowledge, stigma
   - Barriers to service access, social stigma
5. **What makes HIV treatment easier for AYMSM? Please explain.**
   Prompts:
   - Medication-related: ease of use, adherence
   - Personal: perceived need, HIV knowledge
   - Peer and family influence, societal attitudes, healthcare access
6. **What makes HIV treatment difficult for AYMSM? Please explain.**
   Prompts:
   - Medication-related: side effects, adherence
   - Under 18, lack of knowledge, stigma, mental health
   - Family rejection, discrimination
7. **In your opinion, how does family affect HIV prevention among AYMSM?**
   - Positive or negative influence on HIV testing and PrEP use
8. **How does family affect HIV treatment for AYMSM living with HIV?**
   Prompts:
   - Support or pressure regarding care or behavior
   - Influence on risk behaviors (e.g., unsafe sex)
9. **How does family affect the mental health of AYMSM?**
   Prompts:
   - Mental health challenges AYMSM may face
   - Family impact on mental well-being, especially among those living with HIV

**Part 2: Perspectives on a Status-Neutral HIV mHealth App**

*(Suggested time: 15 minutes)*

Imagine a mobile app designed to support AYMSM in both HIV prevention and treatment. This is a **status-neutral intervention**, meaning users can access content tailored to their needs regardless of their HIV status:

- For those unaware of their HIV status: support for HIV testing
- For HIV-negative youth: prevention tools and PrEP support
- For HIV-positive youth: treatment support

**a. Acceptability of a Status-Neutral mHealth App**

1. **What do you think about this idea?**
   Prompts: Do you think AYMSM would use this app? Why or why not?
2. **What are some disadvantages of this combined app compared to separate apps for HIV-negative and HIV-positive youth?**
   Prompts:
   - Concerns for each group
   - Potential to increase or reduce HIV/LGBT stigma
   - Strategies to reduce stigma in the app design
3. **What are some advantages of this combined app compared to separate apps?**
   Prompts: Benefits for both HIV-negative and HIV-positive groups

**b. Expectations for the App**

1. **Besides HIV prevention and sexual health, what other areas should the app address for AYMSM?**
   Prompts:
   - What do you see AYMSM frequently asking about?
   - Mental health? Coming out to family/friends? Community support?
   - Other health issues (e.g., skincare, fitness)?
2. **If the app contains both prevention and treatment content, how should it guide users to the right information based on their HIV status?**
   Prompts:
   - Should these be displayed together or separately?
   - How to present the content in a way that is comfortable and stigma-free?

**Part 3: Feedback on Videos and Messages for the App**

*(Suggested time: 30 minutes)*

We plan to include videos and short messages in the app. These messages will be sent periodically to users via the app. We would like your feedback on the content.

The interviewer will show the participant a list of key message/video topics by ID and category, ask for feedback on each, then ask a general question about each group of messages.

1. **How relevant are these topics to AYMSM?**
   Prompts: What should be added to each group of content (e.g., healthcare)?
2. **Do you prefer text messages or videos? Why?**
3. **How often do you think messages or videos should be sent?**

**Thank you very much for your participation. We greatly value your insights!**
