# Supplementary material for: Cultural Adaptation of Together+, a Status-Neutral mHealth Intervention to Improve HIV Prevention and Care for Adolescent and Young Men Who Have Sex With Men in Vietnam: Protocol for a Co-Design Study
Source: JMIR Res Protoc. 2025 Sep 23;14:e73895. doi: 10.2196/73895 (PMC12504892; doi:10.2196/73895)
Supplement: Multimedia Appendix 2 [file resprot_v14i1e73895_app2.docx]

**Group Discussion Guide – Theater Testing**

**Moving towards a status-neutral approach to improve HIV testing, prevention and care for adolescent and young men who have sex with men in Vietnam: adaptation of an evidence-based mobile health intervention**

**INTRODUCTION**

**Part 1: General Introduction and Objectives**

Thank you for agreeing to meet with me today. My name is [introduce yourself and your role]. I’m working with a research team from Hanoi Medical University. We are designing a status-neutral mobile health intervention to support HIV prevention and treatment among adolescent and young MSM in Vietnam.

We have developed an alpha version of the app and would like to share the design with you. We hope to receive your feedback to make the app more user-friendly, engaging, and effective.

Everything you say today will be kept confidential by the research team. The discussion will be audio recorded and later transcribed, but your name or any identifying information will not be linked to what you say. The recordings will be deleted after analysis is complete.

There are no right or wrong answers. I may sometimes ask you to elaborate, but you can skip any question you're not comfortable answering. Occasionally, I may move on to another question to ensure we cover all topics.

**Group Discussion Rules:**

- Please silence your phones.
- Take turns speaking and avoid interrupting others.
- Keep today’s discussion and others’ opinions confidential.
- Be respectful of all participants and their views.

Do you have any questions before we begin?

**Theater Testing Objectives**

- Present the app’s key features and content to participants and allow group-level interaction with the alpha prototype.
- Assess feasibility and acceptability of the core features and content.
- Collect feedback on functionality, content, user interface, and ease of use to inform app revisions.

**Part 2: Warm-Up**

- Facilitator assigns numbers to participants.
- Facilitator starts recording.
- Facilitator asks participants to briefly introduce themselves:

1. How old are you?
2. Which area of Hanoi do you live in?
3. What is your current job?

**Part 3: Presenting Key App Features and Content**

**App Overview**

Facilitator gives a brief overview:

Imagine a mobile app designed to help adolescent MSM prevent and treat HIV. This is a status-neutral intervention — meaning it serves all users regardless of HIV status.

- For those unaware of their status: supports HIV testing
- For HIV-negative users: supports prevention and PrEP
- For HIV-positive users: supports treatment
  We are now testing the first version and would love your feedback.

**App Demonstration and Group Discussion**

Facilitator presents the app prototype on a screen and walks through each core feature, explaining what the feature does and how it works. After each, the facilitator asks:

- What do you think of this feature?
  - Prompt: Are the details provided sufficient? What content would you add?
- How useful do you think this is for young MSM?
- Which user group do you think will use this feature?

| **#** | **Feature** | **Description and Purpose** |
| --- | --- | --- |
| 1 | HIV Risk Assessment | Users answer questions to assess their HIV risk and receive tailored prevention suggestions. |
| 2 | PrEP Screening | Questionnaire to assess PrEP eligibility (based on Ministry of Health guidelines). |
| 3 | PEP Screening | Questionnaire to assess PEP eligibility based on exposure, timing, and partner status confidence. |
| 4 | HIV Testing Frequency Suggestions | Recommends testing frequency (every 3 or 6 months) based on behavior and STI/substance use history. |
| 5 | Comparing HIV Testing Methods | Information about different HIV tests and suggests suitable options based on user preferences (location, sample method, cost, wait time, etc.). |
| 6 | Testing Planning | Helps users schedule an HIV test with reminders and save test history in the app. |
| 7 | Reminders | Customizable reminders for testing or medication via email, SMS, or app notification. |
| 8 | Ordering Supplies | Allows users to order free items like condoms, lube, and test kits. |
| 9 | Clinic Mapping | Provides addresses and basic info on clinics for HIV testing, PrEP, and treatment in Hanoi. |
| 10 | Info Library and FAQs | Provides essential info and FAQs on HIV risk, testing, treatment, PrEP, condom/lube use. |
| 11 | Text and Video Messages | Sends short, periodic messages in video or text based on users’ risk group. |

**Part 4: Additional Feedback**

- Are there other features you think would benefit young MSM?
  (e.g., chat with a bot or counselor? Connect with the community? Telehealth? Gamification? Newsfeed?)
- Any suggestions for how to present content differently for HIV-negative vs. HIV-positive users?
  - Separate or combined content?
  - How should messages be tailored by HIV status?
- Any suggestions to improve **security and privacy** in the app?

**Thank you very much for participating in this interview. We truly value your input!**

**END**
