# Supplementary material for: Cultural Adaptation of Together+, a Status-Neutral mHealth Intervention to Improve HIV Prevention and Care for Adolescent and Young Men Who Have Sex With Men in Vietnam: Protocol for a Co-Design Study
Source: JMIR Res Protoc. 2025 Sep 23;14:e73895. doi: 10.2196/73895 (PMC12504892; doi:10.2196/73895)
Supplement: Multimedia Appendix 3 [file resprot_v14i1e73895_app3.pdf]

CIPHER
Paediatric HIV
Matters
IAS

Collaborative Initiative for Paediatric HIV Education and Research (CIPHER)

Research Grant Programme 2024

Full Proposal Review and Comment Report

Application ID: 163

Applicant	Xuan Binh Minh Nguyen
Applicant's institution	Hanoi Medical University
Country of Applicant's institution	Vietnam
Project Title	Moving towards a status-neutral approach to improve HIV testing, prevention and care for adolescent and young men who have sex with men in Vietnam: adaptation of an evidence-based mobile health intervention
Mentor	Patrick Sullivan

ID: 163	Reviewer 1	Reviewer 2	Reviewer 3
Significance			
Strengths	• Wants to better understand barriers and facilitators to HIV testing, PrEP use and HIV care among AYMSM in Vietnam, explore perspectives and preferences of AYMSM and other key stakeholders for neutral-status mHealth interventions and adapt the M-Cubed app to develop a status-neutral mHealth intervention to promote HIV testing, PrEP use and HIV care among AYMSM in Vietnam<br>• M-Cubed was developed and tested in the US and found to increase testing and use of PrEP<br>• Focus is on an important population - AYMSM - who have the highest HIV incidence in Vietnam but low rates of HIV testing and PrEP use<br>• Status neutral approach has the ability to reach and engage all AYMSM<br>• mHealth is promising given easy access, privacy, low cost and ability to reach hidden populations<br>• Good cell phone use by MSM in Vietnam<br>• Nice to be building on an existing app but modifying it for AYMSM in Vietnam		• The candidate is addressing a key CIPHER priority head on, and eloquently describes the potential impact of her work.<br>• This CIPHER funding is planned to provide the seed funding needed to do critical development work to then lead to an R01 for an effectiveness implementation trial.
Investigator			

Strengths	• PhD in Health Behaviour from UNC Chapel Hill, completed in 2021<br>• Masters in Epi from UCLA 2015<br>• Medical degree from Hanoi Medical University 2013<br>• Additional training from Washington University in Implementation Research, from JHU in Implementation Science, and in Study Design and Statistics from FHI360<br>• Had a Fogarty Fellowship at UNC Chapel Hill 2022-2023<br>• 25 publications on PubMed, 12 first author<br>• Has received a number of awards and scholarships<br>• Currently a lecturer at Hanoi Medical University<br>• This project builds nicely on the candidate's experience to date - pubs on use of technology by MSM in Vietnam and study of AYMSM findings that almost half had never been tested for HIV and 63% had never used PrEP		• The PI is incredibly strong and has achieved a great deal already, with impressive academic qualifications and papers. She has broad training and experience which really puts her in the perfect position to lead this work. She brings together other collaborators who are required to ensure the project's success (e.g. app developers).
**Approach**			
Strengths	• Nice sequence to the aims<br>• Inclusion of a CAB<br>• Including key stakeholders in Aims 1 and 2<br>• Well detailed plan for recruitment, qualitative data collection, and analysis<br>• Use of the ADAPT-ITT framework for Aim 3<br>• Well described phases for Aim 3 with significant AYMSM input/feedback		• Extremely strong. A clear description and justification are given of the research approach
Weaknesses	• Very minor concern that findings from Aims 1 and 2 could indicate that a status neutral approach isn't wanted but unlikely		
**Innovation**			
Strengths			• mHealth has great potential in Vietnam. The applicant proposes to adapt already validated tools rather than create something new, which is definitely a good approach
**Environment & Resources**			

Strengths	• Partnering with the Institute for Preventive Medicine and Public Health, Hanoi Medical University<br>• Strong letter of support from Prof Le Thi Huong from the Institute for Preventive Medicine and Public Health at Hanoi Medical University<br>• Application includes a long list of resources from Hanoi Medical University, Emory University and UNC Chapel Hill that will be available to her		• The applicant has proven links with the institutions through her research work, lecturing, and academic training, and thus has the potential to make the links a success.
**Mentor**			
Strengths	• Strong mentor who developed the app that Dr. Nguyen wants to adapt<br>• Letter lays out training goals for Dr. Nguyen		• Extremely strong mentor letters
**Overall Impact**			
Strengths	• Exceptionally strong candidate, strong mentor, strong institutional support<br>• well described research methods, research builds on the candidate's prior work and sets her up for an application testing the efficacy of the app		• This is an exceptionally well written and argued proposal, and has clearly been thought through very well. It is of an exceptionally high standard, and has great potential. Information generated should indeed advance research priorities - it is low risk research, in that the applicant is extremely likely to be able to deliver it - and even a null finding (ie a finding that the mHealth app is not appropriate for Vietnam MSM) would be informative.
**Budget**			
	• Yes, the proposed total budget for this project is realistic and reasonable	• Yes, the proposed total budget for this project is realistic and reasonable	• Yes, the proposed total budget for this project is realistic and reasonable
**Comments**			
		• Wonderful proposal with a strong IS approach. It includes a community engagement component to help direct the adaptation of the intervention and throughout the study<br>• I would have liked more information about the intervention she's proposing to adapt and why it was chosen among many other mHealth interventions	• My only comment is whether the PI needs to budget more time for herself, especially in the second year, given she has quite a number of activities to lead and also wishes to write an R01 grant application, which is extremely time consuming but also an appropriate next step for this work and for her career development.
